# Predictors of Response to 24-Week Telaprevir-Based Triple Therapy for Treatment-Naïve Genotype 1b Chronic Hepatitis C Patients

**DOI:** 10.1155/2014/549709

**Published:** 2014-08-14

**Authors:** Hiroshi Abe, Akihito Tsubota, Noritomo Shimada, Masanori Atsukawa, Keizo Kato, Koichi Takaguchi, Toru Asano, Yoshimichi Chuganji, Choitsu Sakamoto, Hidenori Toyoda, Takashi Kumada, Tatsuya Ide, Michio Sata, Yoshio Aizawa

**Affiliations:** ^1^Division of Gastroenterology and Hepatology, Department of Internal Medicine, Jikei University School of Medicine Katsushika Medical Center, 6-41-2 Aoto, Katsushika-ku, Tokyo 125-0062, Japan; ^2^Institute of Clinical Medicine and Research, The Jikei University School of Medicine, Kashiwa, Chiba, Japan; ^3^Department of Gastroenterology and Hepatology, Shinmatsudo Central General Hospital, Matsudo, Chiba, Japan; ^4^Division of Gastroenterology, Department of Internal Medicine, Nippon Medical School Chiba Hokusoh Hospital, Inzai, Chiba, Japan; ^5^Department of Hepatology, Kagawa Prefectural Central Hospital, Takamatsu, Kagawa, Japan; ^6^Department of Gastroenterology, Tokyo Metropolitan Bokutoh Hospital, Sumida-ku, Tokyo, Japan; ^7^Department of Gastroenterology, Nippon Medical School, Graduate School of Medicine, Tokyo, Japan; ^8^Department of Gastroenterology, Ogaki Municipal Hospital, Ogaki, Gifu, Japan; ^9^Division of Gastroenterology, Department of Medicine, Kurume University School of Medicine, Kurume, Fukuoka, Japan

## Abstract

We evaluated the genetic variation in rs8099917, substitutions in core amino acid (aa) 70, and the number of aa substitutions in the interferon sensitivity-determining region (ISDR) on the prediction of sustained virological response (SVR) in treatment-naïve hepatitis C virus (HCV) genotype 1b (G1b) patients. This multicenter study involved 150 Asian treatment-naïve patients infected with HCV G1b who received 12 weeks of telaprevir in combination with 24 weeks of peginterferon-*α*-2b and ribavirin. The baseline and treatment-related factors potentially associated with SVR were determined by multivariate logistic regression analysis. Virological response was analyzed on an intent-to-treat basis. Cessation of the therapy due to adverse effects occurred in only 2 patients, who discontinued the trial at 10 weeks and at 2 weeks due to cerebral infarction and renal impairment, respectively. Among the 150 patients in whom the final virological response was determined, only genotype TT in rs8099917 was identified as a pretreatment predictor (*P* = 7.38 × 10^−4^). Achievement of a rapid virological response (RVR), defined as undetectable HCV RNA at week 4 of treatment, was identified as an after-starting-treatment predictor (*P* = 2.47 × 10^−5^). However, neither a substitution in core aa 70 nor the number of substitutions in the ISDR affected treatment outcome.

## 1. Introduction

Chronic hepatitis C virus (HCV) infection was generally treated with pegylated interferon (PEG-IFN) and ribavirin combination therapy. This treatment provides rates of sustained virological response (SVR, undetectable serum levels of HCV RNA at least 6 months after completion of therapy) of 40–50% among patients with HCV genotype 1 (G1) who have not received previous treatment (treatment-naïve patients) [1–3].

Telaprevir, a first generation orally bioavailable inhibitor of the nonstructural 3/4A HCV protease, shows significantly higher rates of SVR than standard care of PEG-IFN*α* and ribavirin in patients with HCV G1 disease when given for 12 weeks in combination with regimens of PEG-IFN*α* plus ribavirin lasting 12, 24, or 48 weeks [4–6]. Although the SVR rates in patients receiving telaprevir with PEG-IFN*α* and ribavirin (triple therapy) were higher than those in PEG-IFN*α* plus ribavirin (combination therapy) patients, drop-out rates due to increased side effects were also higher in the triple therapy patients [4–6]. Triple therapy is associated with increased risks of anemia, skin lesions, digestive symptoms, hyperuricemia, renal dysfunction, and other side effects compared to the combination therapy, especially among women and older patients [4, 7, 8]. The majority of patients in Japan who are infected with HCV genotype 1b (G1b) are older than the patients in the United States and/or Europe and the frequency of patients who discontinued due to adverse events was reported to be as high as 11.1–16.7% [7–9]. In most previous reports [4–10], the age inclusion criterion limited participants to those who were aged <65 years. Therefore, the safety and efficacy of the triple therapy for patients aged >65 has not been established.

The outcome of the triple therapy in patients who had previously received the combination therapy was largely dependent on the response to the previous therapy [10, 11]. In treatment-naïve patients, the genetic variation near the interleukin-28B (IL-28B) gene and amino acid (aa) substitution in the core 70 (core aa 70) of HCV may be the candidate predictors of the virological response to triple therapy. Although the significance of substitution in the core aa 70 on the outcome of the triple therapy has been reported [8, 9], a considerable number of the patients with a history of the combination therapy were included in these reports. Therefore, the factors predicting SVR in treatment-naïve patients have not been clearly determined.

In the present study, the SVR rate was examined in treatment-naïve patients with HCV G1b who were treated with a regimen consisting of 12 weeks of telaprevir, combined with PEG-IFN*α* and ribavirin, followed by 12 weeks of the PEG-IFN and ribavirin. The significance of genetic variation in rs8099917 near the IL28B gene (rs8099917 genotype), core aa 70, and the number of amino acid substitutions in the interferon sensitivity-determining region (ISDR) on the outcome of triple therapy were then evaluated. In addition, we evaluated the significance of rapid virological response (RVR, serum HCV RNA not detectable within 4 weeks of therapy) [12] in the prediction of SVR and tried to define the patients who were suitable for triple therapy.

## 2. Materials and Methods

### 2.1. Study Population and Study Design

A total of 359 patients, chronically infected with HCV G1b, were treated by triple therapy with telaprevir (Telavic, Tanabe Mitsubishi Pharma, Osaka, Japan), PEG-IFN*α*-2b (PegIntron, MSD, Tokyo, Japan), and ribavirin (Rebetol, MSD) between January 2012 and June 2013. The patients were treated at Jikei University Katsushika Medical Center, Jikei University Kashiwa Hospital, Shinmatsudo Central General Hospital, Nippon Medical School Chiba Hokusoh Hospital, Nippon Medical School Hospital, Kagawa Prefectural Central Hospital, Kurume University School of Medicine, Tokyo Metropolitan Bokutoh Hospital, or Ogaki Municipal Hospital. In these medical institutions, there were no patients who changed the treatment policy by the results of the genetic variation near the IL-28B gene, aa substitution in the core 70, and ISDR of HCV.

Of the 359 patients, 150 had not received previous combination therapy (i.e., they were treatment-naïve) and were included in this analysis (Table 1). The age range of the enrolled patients was 18–75 years. All of the patients satisfied the following criteria. The patients were persistently seropositive for HCV RNA for >6 months and the amount of serum HCV RNA, as determined by a qualitative real-time polymerase chain reaction (RT-PCR) method (COBAS TaqMan HCV test, Roche Diagnostics, Tokyo, Japan), was ≥5 log_10_ IU/mL, which has been defined as a “high viral load,” according to the Japanese criteria [13]. In addition, they had white blood cell counts ≥2000 per cubic millimeter, neutrophil counts ≥1500 per cubic millimeter, hemoglobin levels ≥11 g/dL, and platelet counts ≥70,000 per cubic millimeter, were aged 18–75 years, and had body weights >35 kg at the time of study entry. The presence or absence of cirrhosis was determined by the liver biopsy METAVIR scores [14] within 12 months of enrollment or by an aspartate aminotransferase (AST) to platelet ratio index (APRI) > 2.0, as proposed by Wai et al. [15], at the time of the enrollment. Patients who were positive for the hepatitis B surface antigen or anti-human immunodeficiency virus antibody or who had hepatocellular carcinoma, other liver diseases, psychiatric conditions, or current alcohol consumption levels >20 g per day were excluded from this study.

All patients were scheduled to receive telaprevir (1500–2250 mg per day) combined with weekly subcutaneous injections of PEG-IFN*α*-2b (1.5 *μ*g/kg) and ribavirin (600–1000 mg per day, according to body weight: <60 kg: 600 mg per day; 60–80 kg: 800 mg per day; >80 kg: 1000 mg per day; if the patient's hemoglobin was <13 g/dL at the start of therapy, ribavirin was reduced by 200 mg) for 12 weeks. This was followed by 12 weeks of PEG-IFN*α*-2b plus ribavirin combination therapy. Telaprevir was administered every 8 hours after meals at 500 mg or 750 mg, or every 12 hours after meals at 750 mg or 1125 mg. Ribavirin was orally administered every 12 hours after meals. The patient's attending physicians determined the initial dose of telaprevir (1500 mg per day or 2250 mg per day), based on patient age, gender, body weight, and hemoglobin level. Telaprevir adherence was calculated on the basis of 2250 mg per day. Each drug was appropriately reduced or withdrawn when a serious adverse event developed during the treatment course. In patients who had HCV RNA >3 log_10_ IU/mL at week 4, detectable HCV RNA at 12 week, or a 2 log_10_ IU/mL increase in HCV RNA from the lowest level during therapy, therapy was discontinued because of the extremely low likelihood of achieving SVR and the high risk of developing antiviral resistance.

Virological response was analyzed on an intent-to-treat basis. Virological “relapse” was defined as HCV RNA levels that became undetectable by the end of treatment but became positive again during the follow-up period; viral “breakthrough” was defined as HCV RNA levels that became undetectable during the treatment but became detectable again before the end of the treatment, and “nonvirological response” (NVR) reflected HCV RNA levels that never dropped below the detection level during therapy. We also defined rapid virological response as the absence of detectable HCV RNA 4 weeks after starting treatment.

The study protocol was conducted in accordance with the provisions of the Declaration of Helsinki and Good Clinical Practice guidelines and was approved by the Institutional Review Boards of all participating sites. Written informed consent was acquired from each individual.

### 2.2. Genotyping and Quantification of HCV RNA

The HCV G1b genotype was defined according to the method previously reported by Ohno et al. [16]. The serum HCV RNA concentration was measured at the beginning of therapy and every 4 weeks until 24 weeks after the end of therapy, using the previously described RT-PCR method. The linear dynamic range of the assay was 1.2–7.8 Log_10_ IU/mL, and samples below the level of detection were considered HCV-negative.

### 2.3. Detection of aa Substitutions in Core aa 70 and the ISDR of HCV Genotype 1b

The substitutions in core aa 70 and in the ISDR were determined using a direct sequencing method. Briefly, RNA was extracted from the serum and, after reverse transcription, the substitution in aa 70 was determined according to the method previously reported by Akuta et al. [17]. The “wild-type” aa 70 in the core region is arginine and a “mutant-type” involved a change to glutamine or histidine. Also, aa substitutions in the range of 2209–2248 in the NS5A (the ISDR) were determined using the method of Enomoto et al. [18]. The number of aa substitutions in ISDR was classified into “0-1” and 2 or more.

### 2.4. Genetic Variation Near the IL-28B Gene

Genomic DNA was extracted from whole blood using MagNA Pure LC and the DNA Isolation Kit (Roche Diagnostics). The rs8099917 single nucleotide polymorphism (SNP) near the IL-28B gene [19] was genotyped by RT-PCR using the TaqMan SNP Genotyping Assay and the 7500 Fast RT-PCR System (Applied Biosystems, Foster City, CA, USA). The rs8099917 genotype was classified into 2 categories: TT (major genotype) and non-TT genotype (minor genotype, TG or GG).

### 2.5. Statistical Analysis

Pearson or Mantel-Haenszel chi-square test, Fisher's exact test, or Mann-Whitney test was used to compare frequencies in categorical data or differences in continuous data between groups, respectively. Possible variables contributing to RVR and SVR included baseline and on-treatment features. Variables that reached statistical significance (*P* < 0.05) or marginal significance (*P* < 0.10) in bivariate comparisons were subsequently entered into multivariate logistic regression analysis using forward and backward stepwise selection method to identify significantly independent factors associated with RVR and SVR. *P* values of <0.05 denoted the presence of a statistically significant difference. All statistical analyses were carried out using STATISTICA for Windows version 6 (StatSoft, Tulsa, OK, USA).

## 3. Results

### 3.1. Virological Responses during Triple Therapy

Of the 150 patients, 116 (77.3%) had a RVR. The frequency of undetectable serum HCV RNA was 94.4% at the end of treatment. Of the 150 patients for whom a final virological response was evaluated (either SVR or non-SVR), 131 patients (87.3%) achieved SVR.

### 3.2. Factors Affecting RVR with the Triple Therapy

The frequency of rs8099917 genotype TT (major genotype) (81.0% versus 61.8%; *P* = 0.0195) was significantly higher in patients achieving RVR than in non-RVR patients. Pretreatment serum HCV RNA (6.60 (5.0–7.7) versus 6.95 (5.2–7.8) log_10_ IU/mL; *P* = 0.0153) was also significantly lower in patients achieving RVR than in non-RVR patients. Gender (*P* = 0.0724) and serum alpha-fetoprotein (AFP) (*P* = 0.0655) displayed marginal differences between the RVR and non-RVR patients. Achievement of RVR was not significantly related to the core aa 70 substitutions or to the number of ISDR aa substitutions. In the multivariate logistic regression analysis for elucidating the independent baseline predictive factors for RVR, rs8099917 genotype TT (odds ratio (OR), 0.308; 95% confidence interval (CI), 0.123–0.775; *P* = 0.0116) and pretreatment serum HCV RNA concentrations (OR, 0.293; 95% CI, 0.127–0.675; *P* = 0.0037) were identified (Table 2).

### 3.3. Factors Affecting SVR with the Triple Therapy

The frequency of rs8099917 genotype TT was higher in patients with SVR than non-SVR (85.5% versus 15.8%; *P* = 1.33 × 10^−10^). Serum gamma-glutamyl-transpeptidase concentration was significantly lower in SVR patients than in non-SVR individuals (*P* = 0.0459). Serum albumin (*P* = 0.0968) and fasting plasma glucose concentrations (*P* = 0.0549) demonstrated only marginal differences. The substitutions in core aa 70 and the number of substitutions in the ISDR did not predict SVR. In the multivariate logistic regression analysis for elucidating pretreatment independent predictive factors for SVR, rs8099917 genotype TT (OR, 0.071; 95% CI, 0.015–0.337; *P* = 0.0007) alone was identified. In addition, achievement of RVR (83.2% versus 36.8%; *P* = 2.47 × 10^−5^) after starting treatment was a significant predictor of SVR (Table 3).

### 3.4. Influence of the Substitution in Core aa 70 and the Substitution Number in ISDR on SVR in Patients with rs8099917 Genotype TT or Non-TT

Among the 150 patients, 115 had an rs8099917 TT genotype and 35 had a non-TT (TG or GG) genotype. Of the 115 patients who had genotype TT, 74 had “wild-type” amino acids, whereas 41 had “non-wild-type” aa in the core aa 70 of the HCV isolate. Of the 35 patients who had non-TT (TG or GG) genotype, 23 were “wild-type,” whereas 12 had a “non-wild-type” core aa 70 genotype.

Similarly, among the 115 patients having genotype TT, 87 patients had a “0-1” number of amino acid substitutions, whereas 28 had “2≤” number of ISDR aa substitutions. In the 35 patients with a non-TT genotype, 27 patients had “0-1” and 8 had “2≤” number of ISDR aa substitutions.

**Figure 1:**
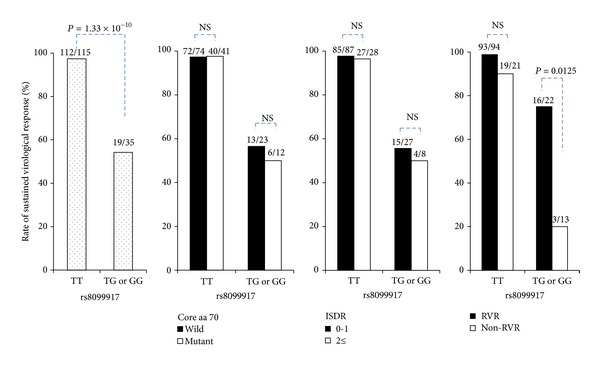
According to the genetic variation in rs8099917, near the IL28B gene, a significantly higher proportion of patients with the TT genotype showed a sustained virological response (SVR) than did patients with the TG or GG genotype. In contrast, based on the amino acid substitutions in the core region (amino acid 70) and interferon sensitivity-determining region (ISDR), there was no significant association between the SVR rate and these substitutions, irrespective of the rs8099917 genotype. Furthermore, the SVR rate was 98.9% in patients with the rs8099917 TT genotype who achieved rapid virological response (RVR), whereas the SVR rate was 23.1% among patients with the TG or GG rs8099917 genotype who did not achieve RVR.

The rates of SVR in these patients are illustrated in Figure 1. Neither the substitution in aa 70 in the HCV core region nor the number of aa substitutions in the ISDR impacted the prediction of SVR, regardless of the rs8099917 genotype.

### 3.5. Significance of RVR on Achieving SVR in Patients with an rs8099917 Non-TT (TG or GG) Genotype

In the 94 patients carrying an rs8099917 TT genotype and achieving RVR, the SVR rate was 98.9% (93/94), whereas it was 90.5% (19/21) in patients carrying genotype TT, but not achieving RVR. In contrast, the SVR rate was only 23.1% (3/13) in patients who had the rs8099917 genotype non-TT (TG or GG) without achieving RVR, whereas it was 72.7% (16/22) in patients achieving RVR (*P* = 0.0125) (Figure 1).

### 3.6. Safety and Characteristics of the Patients Who Did Not Achieve SVR

Of the 150 patients who had a defined final virological response, only 19 (12.7%) were classified as non-SVR: 3 (15.8%) “NVR”; 10 (52.6%) “relapse”; and 6 (31.6%) “breakthrough.” Three patients were carrying genotype TT, whereas 16 had a non-TT genotype. Cessation of the therapy due to adverse effects occurred in only 2 patients who were carrying genotype TT; these patients discontinued the therapy at 2 weeks and at 10 weeks due to cerebral infarction and renal impairment, respectively. The remaining 1 patient who was carrying genotype TT finished the scheduled treatment, but the HCV RNA reappeared 4 weeks after the end of the therapy. These 3 genotype TT patients were classified as having relapses.

### 3.7. Comparison of SVR Rates, according to rs8099917 Genotype and the Existence of Cirrhosis, between Patients Aged ≤65 Years and Those Aged >65 Years

**Figure 2:**
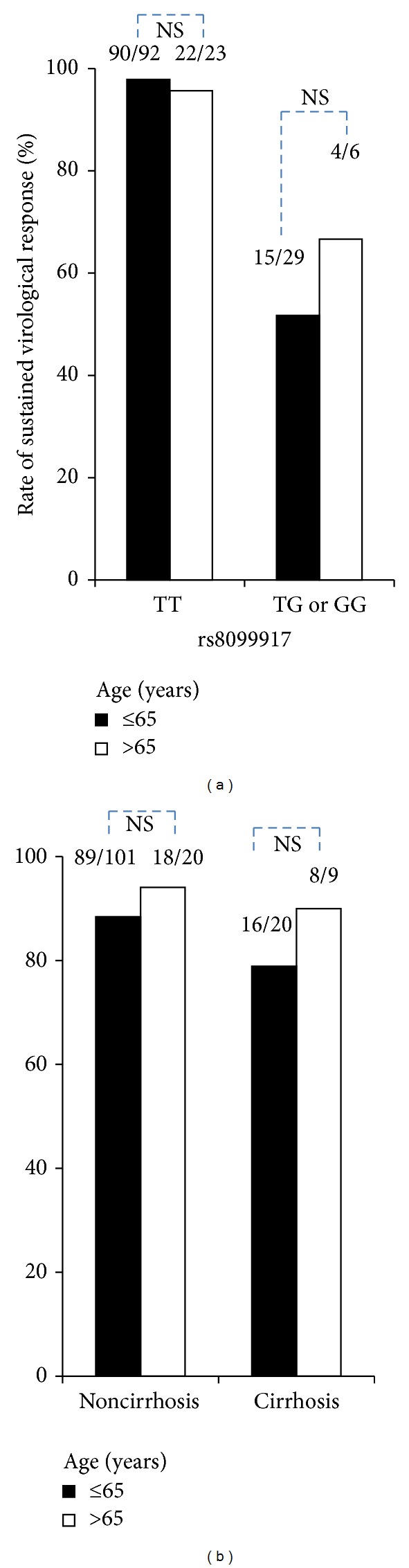
There was no significant relation between sustained virological response rate and age. The patients older than 65 years had hemoglobin levels similar to those aged ≤65 years.

Among the 150 patients, 29 (19.3%) were aged >65 years. The frequency of the non-TT genotype (TG or GG) rs8099917 in the patients in both age groups was similar. However, the frequency of cirrhosis in patients aged over 65 years (9/29; 31.0%) was higher than that in the younger patients (20/121; 16.5%). The SVR rate in the older (aged >65 years) patients was similar to that in those aged ≤65 years, regardless of the rs8099917 genotype or the existence of cirrhosis (Figure 2). In 29 patients aged >65 years, the frequencies of the major side effect were as follows: anemia occurred in 28 (96.6%), elevation of serum uric acid in 20 (69.0%), skin rashes in 18 (62.1%), headache in 15 (51.7%), nausea in 11 (37.9%), and elevation of serum creatinine in 10 (34.4%). The frequencies were similar to those of the ≤65 years patients. Cessation of the therapy due to adverse effects occurred in only 1 (3.4%) patient; this patient discontinued the therapy at 10 weeks due to renal impairment (data not shown).

## 4. Discussion

Genetic variation near the IL28B gene and the substitutions in core aa 70 of HCV G1b have been suggested to be predictive of virological outcomes for triple therapy. However, in the present study of treatment-naïve patients, none of the viral factors (substitution of core aa70 or number of substitutions in the ISDR) or the host factors [12, 20], except for the rs8099917 genotype, were observed as predictors of 24-week triple therapy efficacy.

In the previous study, IL28 SNP genotype had a limited impact on SVR rates with triple therapy in treatment-experienced patients [21], and the strength of association between IL28B genotype and treatment outcome was attenuated in the triple therapy arms compared to the combination therapy arm [22].

In this study, the rs8099917 genotype displayed a striking influence on the outcome of triple therapy, along with “achievement of RVR.” In the patients carrying the rs8099917 genotype TT, even if RVR was not achieved, the SVR rate was as high as 90%. In contrast, the SVR rate was only 23.1% for patients with the TG or GG genotype who did not achieve RVR but reached 72.7% when RVR was achieved. Therefore, “achievement of RVR” was particularly important for SVR in patients with a minor (non-TT) rs8099917 genotype. From these findings, rs8099917 genotyping should be examined in treatment-naïve patients and the patients carrying the TT genotype should be considered for triple therapy. On the other hand, in patients with a TG or GG genotype, the introduction of the triple therapy may be recommended, but extension of treatment period or cessation of the therapy should be considered if HCV RNA remains detectable following 4 weeks of therapy.

Substitutions in the core aa 70 and the number of substitutions in the ISDR of HCV G1b were reported to be important predictors of combination therapy efficacy, and these findings are gaining consensus [12, 23]. In previous reports on the triple therapy, the rs8099917 genotype and the presence of a substitution in the core aa 70 were repeatedly identified as predictors of SVR in patients with HCV G1b infection [8, 9]. However, our study on treatment-naïve patients revealed that core aa 70 substitutions do not predict the achievement of SVR. This discrepancy may be explained due to the differences in the study populations and the inclusion criteria of drop-out patients. Akuta et al. [9] reported that their study population contained both treatment-naïve patients and patients with a history of interferon-based therapy. In addition, 25% of their study population was treated with triple drugs for 12 weeks alone, without the follow-up PEG-IFN plus ribavirin combination therapy. Similarly, Chayama et al. [8] reported that their study population contained not only treatment-naïve patients but also combination therapy experienced patients. In addition, their study included patients who received a very short duration of the triple therapy. Therefore, therapy duration variability and the difference of study population from our study may have had an impact on their study outcomes.

All of our patients were in the intent-to-treat with the 24-week triple therapy and only 6 patients (4.0%) were disrupted. This may lead to our conclusion that substitutions in the core aa 70 did not impact treatment outcomes in treatment-naïve, HCV G1b-infected patients. However, the relatively small number of patients in our study may limit these conclusions; the results should be verified by a larger-scale study.

In our study, the RVR and SVR rates were 80.2% and 87.3%, respectively; these rates tend to be higher than those reported previously [4, 5]. One of the reasons for the higher SVR rate may be the comparatively high frequency (76.7%) of the TT genotype in our cohort. Another reason for higher SVR rate was the extremely low prevalence of drop-outs, owing to the aggressive management of adverse effects and careful adjustment of the dosage of triple drugs. The drop-out rate in the present study was only 1.3% (2 of 150 patients), as compared to 11.1–16.7% in other Japanese studies [7, 9, 24]. Although it is the small number of cases (only 9 of 150 patients), detailed search for aa substitutions in the HCV NS3 protease domain may be required for the care of patients who are classified as NVR or breakthrough, because the relationship between aa substitutions in this domain and resistance to NS3-4A protease inhibitors has been documented [25, 26].

In previous studies [4–10], the treatment of elderly patients with triple therapy is approached with caution and the inclusion criteria for clinical trials are usually set to maximum age of 65 years. Although differences in efficacy and in the frequency and severity of side effects between the patients aged >60 and those aged ≤60 years have not been observed [24], the safety and the efficacy of the treatment in patients aged >65 years has not been adequately demonstrated. However, there is a need for an effective antiviral therapy for older patients in Japan because of the large numbers of older HCV G1 patients. Thus, we included a small number of patients aged >65 years in the present study. The SVR rate of older (>65 years) patients seemed to be similar to that in those aged ≤65 years and there was not a notable increase in the number of drop-outs. Our finding that adherence to the telaprevir or ribavirin dose schedule did not clearly affect the achievement of SVR suggests that continuation of the scheduled length of therapy may be far more important for achieving SVR than maintaining a particular drug dosage. Moreover, the efficacy of triple therapy for the minor rs8099917 genotype or the presence of cirrhosis was similar between patients aged >65 years and those aged ≤65 years. From these observations, we propose that patients aged >65 years can be treated with triple therapy if the drug dosages are adequately regulated.

## 5. Conclusions

In conclusion, for treatment-naïve, chronic HCV G1b-infected patients, the rs8099917 genotype was a significant factor predicting a successful virological effect of the triple therapy; a substitution in core aa 70 and the number of substitutions in the ISDR did not impact treatment outcomes. In addition, achievement of RVR after starting treatment was exceedingly important to accomplish SVR in patients carrying the rs8099917 non-TT genotype. Thus, the patient's rs8099917 genotype should be determined before starting triple therapy.

## Figures and Tables

**Table 1:** Profile and laboratory data at the start of telaprevir-based peginterferon plus ribavirin combination therapy in patients infected with hepatitis C virus genotype 1b.

Demographic data	
Number of patients	150
Gender (male/female)	82/68
Age (years)	58 (18–75)
Body weight (kg)	61.55 (41.2–115.8)
Body mass index (kg/m^2^)	23.4 (17.3–37.8)
Absence or presence of cirrhosis∗	121/29
(noncirrhosis/cirrhosis)	
Histological fibrosis of liver (F0/1/2/3/4/ND)	3/36/28/13/11/59
Genetic variations	
rs8099917 (TT/TG or GG)	115/35
Amino acid substitutions in the HCV genotype 1b	
Core amino acid substitution 70	97/53
(wild-type/mutant-type)∗∗	
Number of amino acid substitutions in ISDR (0-1/2≤)	114/36
Laboratory data	
HCV-RNA (log_10_⁡IU/mL)	6.7 (5.0–7.8)
White blood cells (/*μ*L)	5200 (2000–8700)
Hemoglobin (g/dL)	14.2 (11.0–17.5)
Platelets (×10^4^/*μ*L)	17.1 (7.0–35.3)
Aspartate aminotransferase (IU/L)	45 (13–221)
Alanine aminotransferase (IU/L)	49 (13–305)
Gamma-glutamyl-transpeptidase (IU/L)	40 (12–427)
Albumin (g/dL)	4.1 (3.3–4.7)
Fasting low density lipoprotein-cholesterol (mg/dL)	97.5 (21–194)
Fasting plasma glucose (mg/dL)	101 (74–210)
Homeostasis model assessment-insulin resistance	2.15 (0.68–13.45)
Alpha-fetoprotein (ng/mL)	4.5 (1–235)
Treatment	
Initial dose of PEG-IFN (*μ*g/kg)	1.50 (0.94–1.94)
Initial dose of RBV (mg/kg)	11.5 (6.6–14.0)
Initial dose of TVR (1500/2250 mg)	59/91
Initial dose of TVR (mg/kg)	30.6 (16.7–51.1)

PEG-IFN: peginterferon; RBV: ribavirin; TVR: telaprevir; HCV: hepatitis C virus; ISDR: interferon-sensitivity determining region; ND: not detected.

∗Determined by the liver biopsy METAVIR scores within 12 months of enrollment or by an aspartate aminotransferase to platelet ratio index (APRI) >1.5.

∗∗“Wild-type” (arginine) or “mutant-type” (glutamine or histidine).

Data expressed as number of patients or median (range).

**Table 2:** Patient characteristics at the start of triple therapy for hepatitis C virus genotype 1b, according to achievement of a rapid virological response.

	RVR	Non-RVR	RVR versus non-RVR (1: non-RVR/2: RVR)			
			Univariate analysis	Multivariate analysis		
			*P* value	OR	95% CI	*P* value
Pretreatment factors						
Demographic data						
Number of patients	116	34				
Gender (male/female)	68/48	14/20	0.0724	0.462	0.200–1.065	0.0697
				(1: male/2: female)		
Age (years)	58 (18–75)	57 (29–74)	0.8152			
Body weight (kg)	61.8 (41.2–101.6)	60.1 (44.1–115.8)	0.2956			
Body mass index (kg/m^2^)	23.4 (18.1–34.3)	23.4 (17.3–37.8)	0.6846			
Absence or presence of cirrhosis∗	94/22	27/7	0.8331			
(noncirrhosis/cirrhosis)						
Histological fibrosis of liver (F0/1/2/3/4/ND)	2/26/24/11/8/45	1/10/4/2/3/14				
Genetic variations						
rs8099917 (TT/TG or GG)	94/22	21/13	0.0195	0.308	0.123–0.775	0.0116
				(1: TT/2: TG or GG)		
Amino acid substitutions in the HCV genotype 1b						
Core amino acid substitution 70∗∗	74/42	23/11	0.6793			
(wild-type/mutant-type)						
Number of amino acid substitutions in ISDR (0-1/2≤)	85/31	29/5	0.1490			
Laboratory data						
HCV-RNA (log_10_⁡IU/mL)	6.60 (5.0–7.7)	6.95 (5.2–7.8)	0.0153	0.293	0.127–0.675	0.0037
				(by 1.0 log_10_⁡IU/mL)		
White blood cells (/*μ*L)	5300 (2000–8700)	4790 (3290–7900)	0.2152			
Hemoglobin (g/dL)	14.1 (11.4–17.5)	14.45 (11.0–17.2)	0.5063			
Platelets (/*μ*L)	16.9 (7.0–35.3)	17.35 (7.0–28.8)	0.8858			
Aspartate aminotransferase (IU/L)	42 (13–221)	51.5 (20–135)	0.2394			
Alanine aminotransferase (IU/L)	49 (13–305)	51 (25–169)	0.5716			
Gamma-glutamyl-transpeptidase (IU/L)	37 (12–427)	44.5 (12–359)	0.3205			
Albumin (g/dL)	4.1 (3.3–4.7)	4.0 (3.3–4.7)	0.5075			
Fasting low density lipoprotein-cholesterol (mg/dL)	99 (21–194)	87.5 (58–133)	0.1850			
Fasting plasma glucose (mg/dL)	101 (74–215)	97.5 (80–158)	0.4837			
Homeostasis model assessment-insulin resistance	1.73 (0.68–13.5)	2.24 (0.72–10.1)	0.3215			
Alpha-fetoprotein (ng/mL)	4.4 (1.4–136)	5.35 (1–235)	0.0655	0.978	0.886–1.085	0.6998
				(by 1.0 ng/mL)		
Treatment						
Initial dose of PEG-IFN (*μ*g/kg)	1.50 (1.07–1.82)	1.49 (0.94–1.94)	0.5824			
Initial dose of RBV (mg/kg)	11.3 (6.6–14.1)	11.8 (6.8–13.6)	0.2833			
Initial dose of TVR (1500/2250 mg)	44/72	15/19	0.5161			
Initial dose of TVR (mg/kg)	30.3 (16.7–51.1)	32.1 (19.4–47.1)	0.6470			

After starting treatment factors						
Adherence of PEG-IFN during 4 weeks (%)	100 (50–100)	100 (75–100)	0.4041			
Adherence of RBV during 4 weeks (%)	94.4 (40.4–100)	100 (75–100)	0.1486			
Adherence of TVR∗∗∗ during 4 weeks (%)	83.3 (4.2–100)	84.7 (66.7–100)	0.7318			

PEG-IFN: peginterferon; RBV; ribavirin; TVR: telaprevir; HCV: hepatitis C virus; ISDR: interferon-sensitivity determining region; RVR: rapid virological response.

∗Determined by the liver biopsy METAVIR scores within 12 months of enrollment or by an aspartate aminotransferase to platelet ratio index (APRI) >1.5.

∗∗“Wild-type” (arginine) or “mutant-type” (glutamine or histidine).

∗∗∗Calculated on the basis of 2250 mg/day.

Data expressed as number of patients or median (range).

**Table 3:** Background characteristics of hepatitis C virus genotype 1b patients, based on their achieving sustained virological response.

	SVR	Non-SVR	SVR versus non-SVR (1: non-SVR/2: SVR)			
			Univariate analysis	Multivariate analysis		
			*P* value	OR	95% CI	*P* value
Pretreatment factors						
Demographic data						
Number of patients	131	19				
Gender (male/female)	74/57	8/11	0.2392			
Age (years)	58 (18–75)	57 (40–68)	0.7495			
Body weight (kg)	61.1 (41.2–115.8)	62.5 (45–92.8)	0.8698			
Body mass index (kg/m^2^)	23.4 (17.3–37.8)	23.3 (17.7–31.9)	0.8321			
Absence or presence of cirrhosis∗	107/24	14/5	0.4095			
(1: noncirrhosis/2: cirrhosis)						
Histological fibrosis of liver (F0/1/2/3/4/ND)	3/32/25/11/9/51	0/4/3/2/2/8				
Genetic variations						
rs8099917 (TT/TG or GG)	112/19	3/16	1.33 × 10^−10^	0.071	0.015–0.337	7.38 × 10^−4^
				(1: TT/2: TG or GG)		
Amino acid substitutions in the HCV genotype 1b						
Core amino acid substitution 70∗∗	85/46	12/7	0.8830			
(wild-type/mutant-type)						
Number of amino acid substitutions in ISDR (0-1/2≤)	100/31	14/5	0.8003			
Laboratory data						
HCV-RNA (log_10_⁡IU/mL)	6.7 (5.0–7.8)	6.8 (5.8–7.6)	0.7495			
White blood cells (/*μ*L)	5300 (2000–8700)	4590 (3290–7900)	0.1117			
Hemoglobin (g/dL)	14.2 (11.0–17.4)	13.6 (11.1–17.5)	0.2681			
Platelets (×10^4^/*μ*L)	17.2 (7.0–35.3)	16.5 (7.6–33.6)	0.9123			
Aspartate aminotransferase (IU/L)	43 (13–221)	60 (22–134)	0.1671			
Alanine aminotransferase (IU/L)	48 (13–305)	56 (20–161)	0.2868			
Gamma-glutamyl-transpeptidase (IU/L)	34.5 (12–272)	82 (14–427)	0.0459	0.973	0.872–1.085	0.6134
				(by 10 IU/L)		
Albumin (g/dL)	4.1 (3.3–4.7)	4.0 (3.3–4.7)	0.0968	8.639	0.875–85.736	0.0617
				(by 1.0 g/dL)		
Low density lipoprotein-cholesterol (mg/dL)	99 (51–194)	82 (58–128)	0.1140			
Fasting plasma glucose (mg/dL)	101 (74–210)	91 (80–116)	0.0549	1.621	0.853–3.082	0.1346
				(by 10 mg/dL)		
Homeostasis model assessment-Insulin Resistance	2.15 (0.68–13.5)	1.98 (0.79–12.6)	0.4320			
Alpha-fetoprotein (ng/mL)	4.5 (1–234.7)	12.5 (2.4–117.5)	0.3595			
Treatment						
Initial dose of PEG-IFN (*μ*g/kg)	1.50 (0.94–1.94)	1.48 (1.22–1.72)	0.5049			
Initial dose of RBV (mg/kg)	11.4 (6.6–14.0)	11.6 (6.8–13.3)	0.9482			
Initial dose of TVR (1500/2250 mg)	50/81	9/10	0.4429			
Initial dose of TVR (mg/kg)	30.1 (16.7–51.1)	32.2 (22.2–38.3)	0.8102			

On-treatment factors						
Treatment						
Adherence to PEG-IFN (%)	100 (12.5–100)	94.4 (8.3–100)	0.2302			
Adherence to RBV (%)	66.7 (25.0–100)	67.7 (7.7–100)	0.7006			
Adherence to TVR∗∗∗ (%)	69.4 (25.0–100)	66.7 (15.5–100)	0.1669			
Early virological response						
Achievement of RVR (yes/no)	109/22	7/12	2.47 × 10^−5^			

PEG-IFN: peginterferon; RBV: ribavirin; TVR: telaprevir; HCV: hepatitis C virus; ISDR: interferon-sensitivity determining region; RVR: rapid virological response.

∗Determined by the liver biopsy METAVIR scores within 12 months of enrollment or by an aspartate aminotransferase to platelet ratio index (APRI) >1.5.

∗∗“Wild-type” (arginine) or “mutant-type” (glutamine or histidine).

∗∗∗Calculated on the basis of 2250 mg/day.

Data expressed as number of patients or median (range).

## References

[1] Manns MP, McHutchison JG, Gordon SC (2001). Peginterferon alfa-2b plus ribavirin compared with interferon alfa-2b plus ribavirin for initial treatment of chronic hepatitis C: a randomised trial. *The Lancet*.

[2] Fried MW, Shiffman ML, Rajender Reddy K (2002). Peginterferon alfa-2a plus ribavirin for chronic hepatitis C virus infection. *The New England Journal of Medicine*.

[3] McHutchison JG, Lawitz EJ, Shiffman ML (2009). Peginterferon alfa-2b or alfa-2a with ribavirin for treatment of hepatitis C infection. *The New England Journal of Medicine*.

[4] McHutchison JG, Everson GT, Gordon SC (2009). Telaprevir with peginterferon and ribavirin for chronic HCV genotype 1 infection. *The New England Journal of Medicine*.

[5] Hézode C, Forestier N, Dusheiko G (2009). Telaprevir and peginterferon with or without ribavirin for chronic HCV infection. *The New England Journal of Medicine*.

[6] McHutchison JG, Manns MP, Muir AJ (2010). Telaprevir for previously treated chronic HCV infection. *The New England Journal of Medicine*.

[7] Kumada H, Toyota J, Okanoue T, Chayama K, Tsubouchi H, Hayashi N (2012). Telaprevir with peginterferon and ribavirin for treatment-naive patients chronically infected with HCV of genotype 1 in Japan. *Journal of Hepatology*.

[8] Chayama K, Hayes CN, Abe H (2011). IL28B but not ITPA polymorphism is predictive of response to pegylated interferon, ribavirin, and telaprevir triple therapy in patients with genotype 1 hepatitis C. *Journal of Infectious Diseases*.

[9] Akuta N, Suzuki F, Hirakawa M (2010). Amino acid substitution in hepatitis C virus core region and genetic variation near the interleukin 28B gene predict viral response to telaprevir with peginterferon and ribavirin. *Hepatology*.

[10] Zeuzem S, Andreone P, Pol S (2011). Telaprevir for retreatment of HCV infection. *The New England Journal of Medicine*.

[11] Muir AJ, Poordad FF, Mchutchison JG (2011). Retreatment with telaprevir combination therapy in hepatitis C patients with well-characterized prior treatment response. *Hepatology*.

[12] Akuta N, Suzuki F, Hirakawa M (2012). Amino acid substitution in HCV core/NS5A region and genetic variation near IL28B gene affect treatment efficacy to interferon plus ribavirin combination therapy. *Intervirology*.

[13] Kumada H, Okanoue T, Onji M (2010). Guidelines for the treatment of chronic hepatitis and cirrhosis due to hepatitis C virus infection for the fiscal year 2008 in Japan. *Hepatology Research*.

[14] Bedossa P, Poynard T (1996). An algorithm for the grading of activity in chronic hepatitis C. *Hepatology*.

[15] Wai C, Greenson JK, Fontana RJ (2003). A simple noninvasive index can predict both significant fibrosis and cirrhosis in patients with chronic hepatitis C. *Hepatology*.

[16] Ohno T, Mizokami M, Wu R (1997). New hepatitis C virus (HCV) genotyping system that allows for identification of HCV genotypes 1a, 1b, 2a, 2b, 3a, 3b, 4, 5a, and 6a. *Journal of Clinical Microbiology*.

[17] Akuta N, Suzuki F, Sezaki H (2005). Association of amino acid substitution pattern in core protein of hepatitis C virus genotype 1b high viral load and non-virological response to interferon-ribavirin combination therapy. *Intervirology*.

[18] Enomoto N, Sakuma I, Asahina Y (1996). Mutations in the nonstructural protein 5A gene and response to interferon in patients with chronic hepatitis C virus 1b infection. *The New England Journal of Medicine*.

[19] Tanaka Y, Nishida N, Sugiyama M (2009). Genome-wide association of IL28B with response to pegylated interferon-α and ribavirin therapy for chronic hepatitis C. *Nature Genetics*.

[20] Everson GT, Hoefs JC, Seeff LB (2006). Impact of disease severity on outcome of antiviral therapy for chronic hepatitis C: lessons from the HALT-C trial. *Hepatology*.

[21] Pol S, Aerssens J, Zeuzem S (2013). Limited impact of IL28B genotype on response rates in telaprevir-treated patients with prior treatment failure. *Journal of Hepatology*.

[22] Holmes JA, Desmond PV, Thompson AJ (2012). Does IL28B genotyping still have a role in the era of direct-acting antiviral therapy for chronic hepatitis C infection?. *Journal of Viral Hepatitis*.

[23] Hayes CN, Kobayashi M, Akuta N (2011). HCV substitutions and IL28B polymorphisms on outcome of peg-interferon plus ribavirin combination therapy. *Gut*.

[24] Furusyo N, Ogawa E, Nakamuta M (2013). Telaprevir can be successfully and safely used to treat older patients with genotype 1b chronic hepatitis C. *Journal of Hepatology*.

[25] Sarrazin C, Zeuzem S (2010). Resistance to direct antiviral agents in patients with hepatitis C virus infection. *Gastroenterology*.

[26] Romano KP, Ali A, Royer WE, Schiffer CA (2010). Drug resistance against HCV NS3/4A inhibitors is defined by the balance of substrate recognition versus inhibitor binding. *Proceedings of the National Academy of Sciences of the United States of America*.

